# Fatal Suicide Attempt with Upadacitinib (Rinvoq^®^) in an Adolescent: A Case Report

**DOI:** 10.3390/reports9010009

**Published:** 2025-12-24

**Authors:** Silviya Stoykova, Ivo Ivanov, Evgeniya Byrzashka, Vasil Atanasov

**Affiliations:** 1 Forensic Toxicology Laboratory, Department of Toxicology, Military Medical Academy, 3, “St. Georgi Sofiyski” Str., 1606 Sofia, Bulgaria; ahidi@chem.uni-sofia.bg (I.I.); ahva@chem.uni-sofia.bg (V.A.); 2 Laboratory of Biocoordination and Bioanalytical Chemistry, Department of Analytical Chemistry, Faculty of Chemistry and Pharmacy, Sofia University “St. Kliment Ohridski”, 1, “James Bourchier” Blvd., 1164 Sofia, Bulgaria; 3 Clinical Toxicology Clinic, Medical University–Pleven, 1, “St. Kliment Ohridski” Str., 5800 Pleven, Bulgaria; byrzashka@abv.bg; 4 Clinical Toxicology Clinic, University Multiprofile Hospital for Active Treatment “Dr. Georgi Stranski”, 8A, “Georgi Kochev” Blvd., 5809 Pleven, Bulgaria

**Keywords:** upadacitinib, JAK inhibitors, suicide, overdose

## Abstract

**Background and Clinical Significance**: Upadacitinib, a selective Janus kinase 1 (JAK1) inhibitor, is increasingly prescribed for autoimmune and inflammatory diseases. Although its therapeutic safety profile is well established, fatal intoxications have not been reported to date. **Case Presentation**: We describe the first fatal case of upadacitinib overdose in a 13-year-old girl. Following ingestion of approximately 600 mg (40 × 15 mg tablets Rinvoq^®^), the patient presented with deep coma, profound bradycardia (~40 bpm) with third-degree atrioventricular block, conduction delay, hypotension, hypothermia, and metabolic acidosis. Laboratory tests showed hyperglycemia (17.8 mmol/L) and only minimal elevations in cardiac biomarkers (CK 57.03 U/L, CK-MB 30.64 U/L, troponin 0.003 ng/mL). Despite advanced resuscitation, the patient succumbed within a few hours. Forensic toxicology revealed extremely high concentrations of upadacitinib, 1.84 µg/mL (~1840 ng/mL) in blood and 70.3 µg/mL in gastric contents, far exceeding reported therapeutic plasma levels (Cmax 36.0 ± 8.8 ng/mL). This case establishes the first reported value for a lethal upadacitinib concentration in humans. The combination of conduction abnormalities, refractory shock, and minimal biomarker changes is consistent with an acute electrophysiological and hemodynamic collapse rather than myocardial infarction. **Conclusions**: The toxicity of upadacitinib in this case is characterized by profound central nervous system depression, severe cardiovascular (electrophysiological and hemodynamic) disturbances, and metabolic abnormalities (acidosis and hyperglycemia). These findings provide essential reference data for clinical and forensic toxicology, highlight the fatal potential of upadacitinib in overdose, and underscore the importance of secure medication storage and pharmacovigilance in households with adolescents.

## 1. Introduction and Clinical Significance

**Figure 1:**
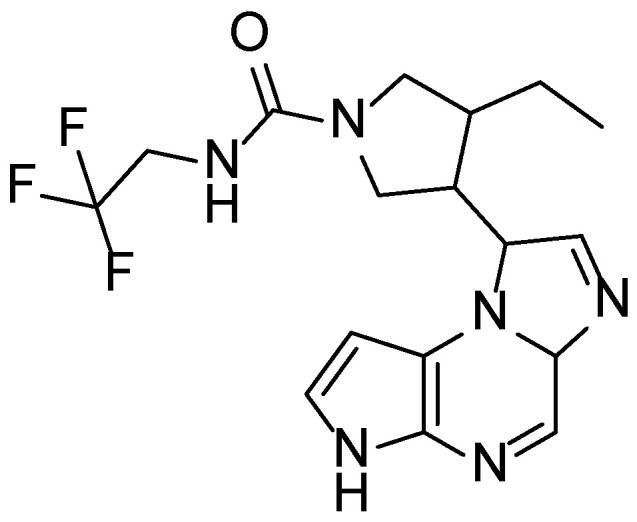
Chemical structure of upadacitinib.

Upadacitinib (Figure 1) is an orally administered, second-generation selective Janus kinase 1 (JAK1) inhibitor developed to improve efficacy while reducing unwanted activity against other JAK isoforms. It is currently available as an extended-release formulation (usually 15 mg once daily) and has received approval for the treatment of a broad spectrum of immune-mediated inflammatory diseases, including rheumatoid arthritis, psoriatic arthritis, ankylosing spondylitis, atopic dermatitis, Crohn’s disease, and ulcerative colitis [1,2,3,4,5,6,7]. By selectively targeting JAK1 within the JAK-STAT pathway, the drug inhibits key pro-inflammatory cytokines such as interleukin-6 (IL-6), interferon-γ (IFN-γ), and granulocyte-macrophage colony-stimulating factor (GM-CSF) [6,8].

Pharmacokinetic studies have shown that upadacitinib is rapidly absorbed, with maximum plasma concentrations reached within 2–4 h after administration. Its terminal elimination half-life ranges between 8 and 14 h, with plasma protein binding of approximately 52%. Biotransformation occurs mainly through cytochrome P450 3A4 (CYP3A4), with a minor involvement of CYP2D6, and both unchanged drug and metabolites are excreted in urine and feces [6,9,10,11]. At steady state, trough concentrations (Cmin) after 15 mg extended-release dosing average 2.8 ± 1.2 ng/mL, whereas peak concentrations (Cmax) reach about 36.0 ± 8.8 ng/mL [9,10,11,12].

Although upadacitinib has demonstrated sustained efficacy and an acceptable safety profile in clinical trials, concerns have arisen about class-specific risks of JAK inhibitors, particularly infections, herpes zoster reactivation, dyslipidemia, and, more recently, cardiovascular and malignancy outcomes [13,14,15,16,17,18,19,20,21,22,23,24,25,26]. However, real-world registries such as BIOBADASER (BIOlogical BADevent Analysis SERies) and RABBIT (Rheumatoid Arthritis: Observation of Biologic Therapy) suggest that this risk is primarily associated with older patients who have pre-existing comorbidities, such as hypertension or diabetes, or who have experienced prior cardiovascular events [27,28]. Importantly, while therapeutic plasma levels are well documented, no published studies have defined toxic or lethal concentrations in humans. This absence of toxicological reference values creates a challenge for forensic interpretation in cases of suspected overdose.

This study presents the first toxicologically confirmed fatal case of intentional ingestion of upadacitinib (Rinvoq^®^) in an adolescent patient. The findings provide an initial benchmark for lethal concentration, underscore the importance of including upadacitinib in forensic toxicology screening, and highlight the broader public health implications of unsupervised pediatric access to potent immunomodulatory drugs prescribed to household members.

## 2. Case Presentation

A 13-year-old female with no chronic illnesses but a history of a prior suicide attempt ingested approximately 40 tablets of Rinvoq^®^ (15 mg each; total dose 600 mg) that had been prescribed to a family member. The act was reportedly triggered by an acute psychosocial conflict. She was discovered unconscious at home and transported to the nearest emergency department.

**Figure 2:**
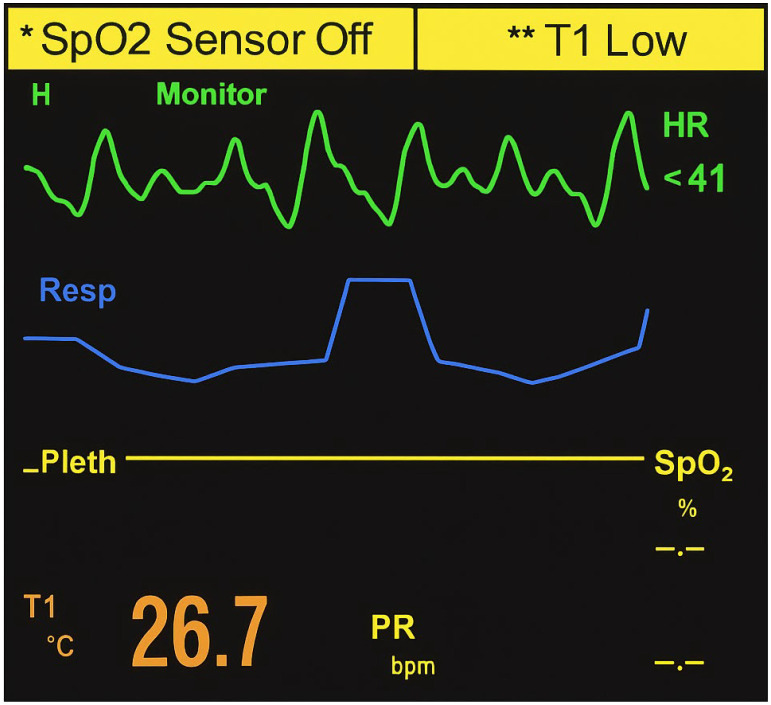
Monitor tracing on admission showing severe sinus bradycardia (~40 bpm) with complete atrioventricular (third-degree) block and markedly widened QRS complexes consistent with bundle branch block. The tracing demonstrates complete atrioventricular dissociation and slow ventricular escape rhythm, indicating acute conduction system failure and cardiogenic shock consistent with toxic depression of cardiac function following massive upadacitinib ingestion.

On admission, she was deeply comatose (Glasgow Coma Scale score of 3/15), cyanotic, and exhibited dilated, sluggish pupils. Monitor tracings revealed marked bradycardia (~40 bpm) with third-degree atrioventricular (AV) block and intraventricular conduction delay consistent with bundle branch block (Figure 2). The tracing demonstrated complete AV dissociation and a slow, wide QRS (QRS complex on electrocardiography) ventricular escape rhythm, consistent with toxic depression of cardiac conduction. These findings correlated clinically with profound hypotension (50/20 mmHg) and hypothermia (26.7 °C), reflecting an acute electrophysiological and hemodynamic collapse. Laboratory testing showed severe metabolic acidosis (pH 7.159, base excess –12.8 mEq/L) and hyperglycemia (17.8 mmol/L). During admission, a short tonic-clonic seizure was observed, and chest auscultation indicated pulmonary edema, consistent with the hypoxic-toxic state. Cardiac biomarkers demonstrated creatine kinase (CK) of 57.03 U/L (reference 30–200 U/L), CK-MB of 30.64 U/L (reference <25 U/L), and troponin of 0.003 ng/mL (reference <0.04 ng/mL). Other paraclinical parameters, including leukocyte count, hemoglobin, creatinine, and electrolytes, were within reference ranges.

Emergency treatment included gastric lavage with 3 L of water, endotracheal intubation, and mechanical ventilation. Pharmacologic interventions comprised atropine, adrenaline (epinephrine), noradrenaline (norepinephrine), dopamine, dobutamine, sodium bicarbonate, methylprednisolone, and valproic acid. A temporary pacemaker was inserted but failed to achieve ventricular capture. Despite repeated resuscitation with adrenaline, atropine, and ephedrine, cardiovascular function could not be restored, and the patient died within 3.5 h of admission.

Urine and gastric contents were collected during hospitalization as part of emergency procedures—urine via catheterization and gastric fluid during gastric lavage. Following death, a full autopsy was performed within 24 h. Internal examination revealed non-specific signs of acute circulatory failure, including visceral congestion and pulmonary edema. Femoral blood was obtained in a clean container without anticoagulants, preservatives or additives during the autopsy. All samples were submitted for comprehensive forensic toxicological analysis.

### Forensic Toxicology Analysis

Initial screening of blood and urine for toxic alcohols (ethanol and methanol) was performed using headspace gas chromatography with flame ionization detection (HS-GC-FID; Agilent 7890B GC, 7697A HS) [29]. Both matrices tested negative.

Subsequently, multi-panel immunoassays (AllTest Biotech Co, Hangzhou China) were applied to screen blood and urine for common drugs of abuse, including cannabinoids, amphetamines, methamphetamines, 3,4-methylenedioxymethamphetamine, cocaine, opiates, methadone, benzodiazepines, barbiturates, and tricyclic antidepressants, with established cut-off concentrations [29]. All results were negative.

General unknown screening of urine was then conducted using gas chromatography-mass spectrometry (GC–MS; Agilent 7890B GC, 5977A MSD) after liquid-liquid extraction (LLE) [29]. Library searches were performed against PMWTox3N, DD2011, and NIST 2011 databases. This analysis revealed only therapeutic agents administered during resuscitation and treatment—piracetam, lidocaine, and methylprednisolone.

Quantitative determination of upadacitinib in blood and gastric contents was performed by high-performance liquid chromatography with diode-array detection (HPLC–DAD; Agilent 1260 Infinity system) using a reversed-phase Zorbax C-18 column (150 mm × 4.6 mm, 5 µm). The mobile phase consisted of 25% acetonitrile in 10 mM triethylamine-phosphate buffer, adjusted to pH 5.2, at a flow rate of 1.0 mL/min. The injection volume was 20 µL and detection was carried out at 230 nm. Calibration was based on an external standard solution of upadacitinib (1.50 µg/mL), prepared from Rinvoq^®^ tablets in methanol-water (50:50, *v*/*v*).

Sample preparation involved protein precipitation of 1 mL of blood or gastric contents with 1.5 mL acetonitrile, followed by addition of 2 mL deionized water and a double LLE with 3 mL ethyl acetate. After vortex mixing and centrifugation (3 min, 3000 rpm), the organic layers were dried over magnesium sulphate, evaporated to dryness under nitrogen, and reconstituted in 250 µL of the mobile phase prior to injection. The overall extraction yield was 98.2%.

**Figure 3:**
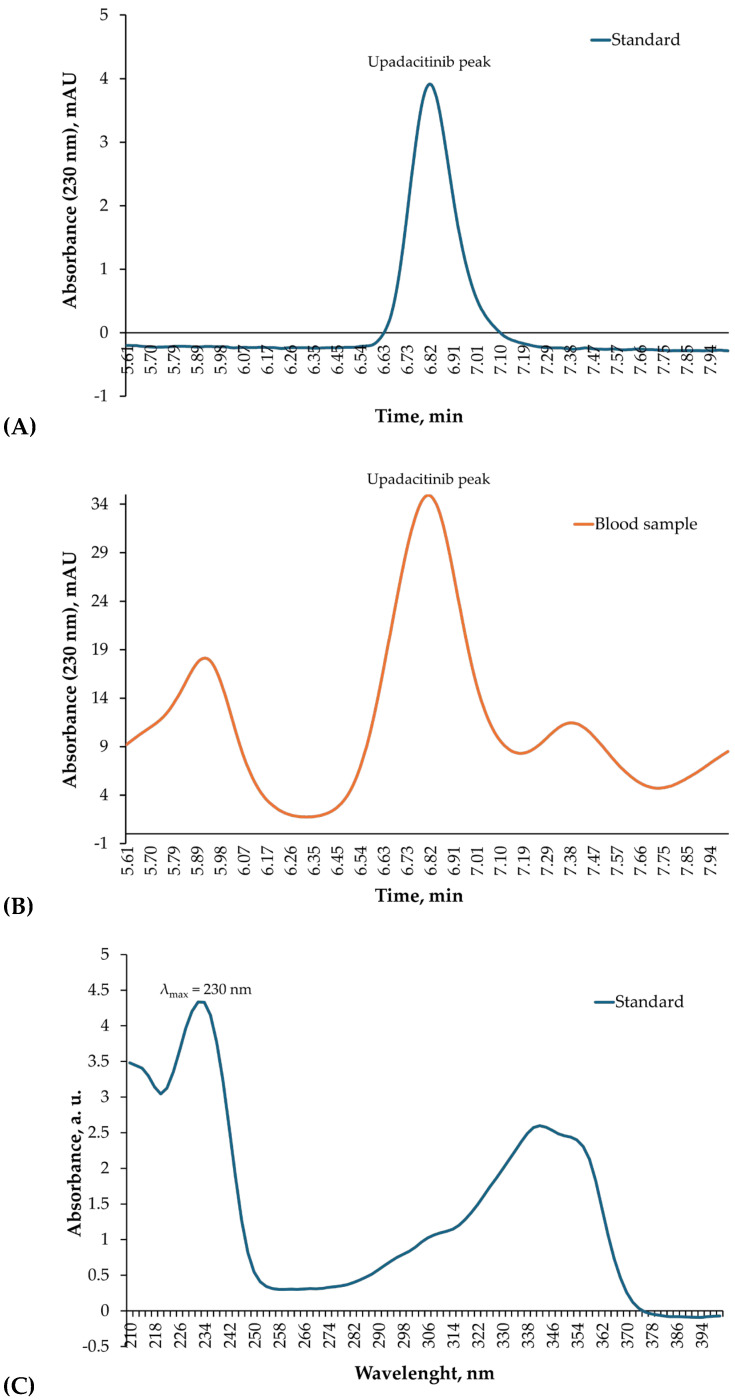

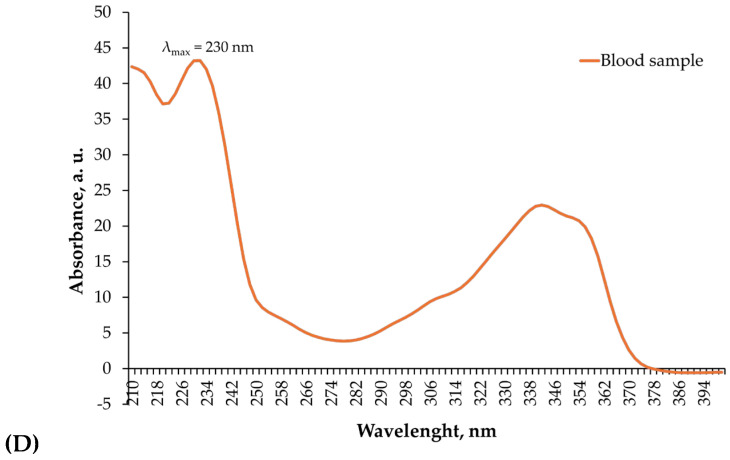
HPLC–DAD identification of upadacitinib in blood. (**A**) Chromatogram of the upadacitinib external standard (1.50 µg/mL) recorded at 230 nm, showing a single sharp peak with a RT of 6.82 min. (**B**) Chromatogram of the patient’s blood sample acquired under identical chromatographic conditions, displaying a corresponding peak at the same RT. (**C**) Diode-array absorption spectra (200–400 nm) of the upadacitinib standard. (**D**) Diode-array absorption spectra (200–400 nm) of the patient’s blood sample peak, demonstrating high spectral concordance with the standard across the analytical wavelength range used for identification.

The chromatographic analysis revealed a distinct upadacitinib peak at a retention time (RT) of 6.82 min, identical to that of the external standard. Diode-array detection confirmed the analyte identity through overlapping UV absorption spectra (200–400 nm) obtained from both the standard and the biological sample (Figure 3A–D). Quantification in blood was performed using manual peak integration with consistent baseline selection, guided by exact RT matching and full spectral concordance with the standard. Minor spectral deviations observed at wavelengths below 220 nm were attributed to matrix-related background absorption and did not affect compound identification, which was based on complete spectral overlap within the 230–350 nm range.

The blood concentration of upadacitinib was determined to be 1.84 µg/mL (~1840 ng/mL), while the gastric contents contained 70.3 µg/mL, indicating massive recent ingestion. These findings provide unequivocal toxicological confirmation of acute lethal overdose.

## 3. Discussion

This case provides the first toxicologically confirmed fatality due to intentional ingestion of upadacitinib. The clinical presentation was characterized by rapid onset of deep coma, severe bradycardia (~40 bpm) with third-degree AV block, and conduction abnormalities resembling bundle branch block, accompanied by profound hypotension and hypothermia. The monitor tracing (Figure 2) demonstrated complete AV dissociation and a wide QRS ventricular escape rhythm, confirming a critical failure of cardiac conduction and severe bradyarrhythmia consistent with toxic cardiodepression. Laboratory investigations revealed severe metabolic acidosis (pH 7.159, base excess −12.8 mEq/L) and marked hyperglycemia (17.8 mmol/L). The observed metabolic acidosis and hyperglycemia are interpreted as manifestations of severe systemic stress and acute toxicity rather than as direct effects of JAK1 inhibition. Cardiac biomarkers, however, showed only minimally elevated CK–MB (30.64 U/L; reference <25 U/L), while CK (57.03 U/L; reference 30–200 U/L) and troponin (0.003 ng/mL; reference <0.04 ng/mL) remained within normal ranges. These findings argue against acute myocardial infarction as the mechanism of death and instead support an acute electrophysiological and hemodynamic collapse induced by massive upadacitinib exposure.

Quantitative toxicological analysis using HPLC–DAD confirmed extremely high concentrations of upadacitinib in the analyzed biological matrices. Chromatographic identification showed a single, well-defined peak at a RT of 6.82 min, identical to that of the standard, and the DAD absorption spectra were fully superimposable, confirming analyte identity (Figure 3A–D). The quantitative results demonstrated 1.84 µg/mL (~1840 ng/mL) of upadacitinib in blood and 70.3 µg/mL in gastric contents.

When compared with reported therapeutic steady-state plasma concentrations (Cmin 2.8 ± 1.2 ng/mL; Cmax 36.0 ± 8.8 ng/mL) [9,10,11,12], the measured blood level exceeded the upper therapeutic limit by more than fifty-fold. Toxicokinetic studies in clinical settings rarely document concentrations above 100 ng/mL, even under intensified dosing regimens for inflammatory bowel disease [23,30]. Accordingly, the present observation provides the first documented human reference value for upadacitinib concentrations associated with a fatal outcome. While extrapolation from a single case must be made with caution, the magnitude of exposure observed here strongly supports a direct causal relationship between extreme systemic drug levels and fatal toxicity.

The clinical manifestations observed here are consistent with the pharmacological profile of upadacitinib. While at therapeutic doses it selectively inhibits JAK1, blocking signaling of pro-inflammatory cytokines such as IL-6, IFN-γ, and GM–CSF [1,2,3,4,5,6,7,8], supratherapeutic exposure likely results in off-target effects that disrupt cardiac conduction and vascular tone. Animal studies with high-dose JAK inhibitors have described disturbances in electrophysiology and autonomic regulation [31,32]. In this case, conduction abnormalities (third-degree AV block, wide QRS complexes) and refractory shock strongly suggest acute cardiotoxicity mediated through such mechanisms. Direct evidence of mitochondrial dysfunction or cytokine-mediated autonomic dysregulation was not available in this case. Therefore, mechanistic considerations are based on pharmacological plausibility rather than direct experimental confirmation. The high concentration of unmetabolized drug in gastric contents further indicates prolonged absorption from the extended-release formulation, which may have contributed to sustained toxicity and the inability of resuscitative efforts to restore circulation.

Comparison with other JAK inhibitors underscores the uniqueness of this case. For tofacitinib, baricitinib, filgotinib, and ruxolitinib, published clinical safety data and post-marketing surveillance have primarily focused on long-term risks, including infections, herpes zoster reactivation, malignancies, major adverse cardiovascular events, and venous thromboembolism [1,2,3,4,5,6,7,13,14,15,16,17,18,19,20,21,22,23,24,25,26,33]. Acute fatalities due to overdose have not been previously described. Case reports of overdose with tofacitinib and baricitinib remain scarce and have generally been managed successfully with supportive care, owing to their shorter half-lives and lack of extended-release formulations. By contrast, in the present case, the ingestion of a massive dose of upadacitinib in an extended-release form produced plasma concentrations far exceeding therapeutic ranges, overwhelming pharmacokinetic elimination capacity and rendering conventional intensive care interventions ineffective.

This case also highlights specific pediatric and public health considerations. Upadacitinib is not broadly approved for patients under 18 years of age, although intensified regimens have been explored in adolescents with severe ulcerative colitis, with favorable outcomes and no reported fatalities [30]. Nevertheless, the availability of potent immunomodulators within households represents a serious risk when unsupervised access by children or adolescents is possible. Intentional ingestion in vulnerable adolescents, as illustrated here, carries a high fatal potential. Secure medication storage and careful risk assessment should therefore be emphasized when prescribing such agents.

From a forensic perspective, the concentration of 1.84 µg/mL reported here represents the first toxicologically confirmed lethal level of upadacitinib in humans and provides an essential reference point for future case interpretation.

A limitation of this study is the use of HPLC–DAD rather than LC–MS/MS for quantitative confirmation. Nevertheless, compound identification was supported by concordant retention time with an external standard, characteristic diode-array absorption spectra, and consistency across multiple biological matrices and the clinical context. In many forensic laboratories, HPLC–DAD remains a validated and routinely applied technique for drug identification and quantification when mass spectrometric instrumentation is not immediately available.

## 4. Conclusions

This case report describes the first toxicologically confirmed fatal intoxication with upadacitinib, a selective JAK1 inhibitor, in a 13-year-old patient who intentionally ingested 600 mg (40 × 15 mg tablets). The clinical presentation was dominated by deep coma, severe bradycardia, third-degree AV block, conduction abnormalities, profound hypotension, hypothermia, and metabolic acidosis. Laboratory analyses revealed hyperglycemia and only minimal changes in cardiac biomarkers (CK, CK-MB, and troponin), findings that argue against acute myocardial infarction as the underlying cause of death. Instead, the constellation of conduction disturbances, refractory shock, and rapid cardiovascular collapse strongly indicates an acute electrophysiological and hemodynamic mechanism of toxicity.

Forensic toxicological analysis provided unequivocal confirmation, with upadacitinib detected in both gastric contents and blood. Quantitative measurements revealed blood concentrations of 1.84 µg/mL (~1840 ng/mL) and gastric content levels of 70.3 µg/mL, far exceeding reported therapeutic plasma concentrations (Cmax 36.0 ± 8.8 ng/mL). While conclusions must be interpreted with caution given the single-case nature of the report, these represent the first documented human reference values for upadacitinib concentrations associated with a fatal outcome and suggest that plasma levels in the µg/mL range are incompatible with survival.

This case underscores the forensic, clinical, and public health significance of upadacitinib overdose. Forensic laboratories should include this agent in routine toxicological screening, particularly in cases of sudden unexplained death among patients with autoimmune or inflammatory conditions. Clinicians should recognize the potential for acute lethality, especially in households with adolescents who may access potent immunomodulators prescribed to family members. Secure storage, patient education, and pharmacovigilance reporting are therefore essential preventive strategies.

By integrating clinical presentation, laboratory findings, and toxicological results, this report provides critical reference data for the interpretation of upadacitinib intoxication and highlights a previously unrecognized fatal toxicity pattern within the class of JAK inhibitors.

## Data Availability

All relevant data are included in the article. Further inquiries can be directed to the corresponding author.

## Abbreviations

The following abbreviations are used in this manuscript:

JAK1	Janus kinase 1 inhibitor
JAK-STAT	Janus kinase-signal transducers and activators of transcription
IL-6	interleukin-6
IFN-γ	interferon-γ
GM-CSF	granulocyte-macrophage colony-stimulating factor
CYP3A4	cytochrome P450 3A4
CYP2D6	cytochrome P450 2D6
Cmin	the lowest concentration of a drug in the blood
Cmax	the highest (peak) concentration of a drug in the bloodstream
JAK	Janus kinase
BIOBADASER	BIOlogical BADevent Analysis SERies
RABBIT	Rheumatoid Arthritis: Observation of Biologic Therapy
AV	atrioventricular
CK	creatine kinase
CK-MB	creatine kinase-MB
HS-GC-FID	headspace gas chromatography with flame ionization detection
GC	gas chromatography
HS	headspace
GC-MS	gas chromatography-mass spectrometry
MSD	masspectral detector
LLE	liquid-liquid extraction
HPLC-DAD	high-performance liquid chromatography with diode-array detection
RT	retention time
